# Functional and Genomic Characterization of *Serratia quinivorans* NFX21 and *Pseudomonas thivervalensis* NFX104, Novel Biocontrol Agents Against *Botrytis cinerea*

**DOI:** 10.3390/plants15071052

**Published:** 2026-03-29

**Authors:** Sara Tedesco, Filipa T. Silva, Margarida Pimenta, Frédéric Bustos Gaspar, Marta Nunes da Silva, Maria Teresa Barreto Crespo, Francisco X. Nascimento

**Affiliations:** 1 iBET—Instituto de Biologia Experimental e Tecnológica, 2780-157 Oeiras, Portugal; sara.tedesco@ibet.pt (S.T.); filipa.silva@ibet.pt (F.T.S.); margarida.pimenta@ibet.pt (M.P.); fgaspar@ibet.pt (F.B.G.); tcrespo@ibet.pt (M.T.B.C.); 2 ITQB NOVA—Instituto de Tecnologia Química e Biológica António Xavier, Universidade Nova de Lisboa, 2780-157 Oeiras, Portugal; 3 CBQF—Centro de Biotecnologia e Química Fina—Laboratório Associado, Escola Superior de Biotecnologia, Universidade Católica Portuguesa, 4169-005 Porto, Portugal; mansilva@ucp.pt

**Keywords:** *Botrytis cinerea*, *Serratia*, *Pseudomonas*, biocontrol, antifungal, gray mold

## Abstract

*Botrytis cinerea*, the causative agent of gray mold, is a major fungal pathogen affecting a wide range of economically important crops. To identify sustainable alternatives to chemical fungicides, this study characterized the biocontrol potential of two bacterial strains, *Serratia quinivorans* NFX21 and *Pseudomonas thivervalensis* NFX104, through genomic analysis and functional assays targeting key stages of fungal growth and plant infection. The NFX21 and NFX104 strains significantly inhibited *B. cinerea* mycelial growth (~35%) and strongly suppressed conidial germination with performances comparable to the reference biocontrol strain *Bacillus amyloliquefaciens* QST 713. In tomato detached-leaf and whole-plant pot assays, application of NFX21 and NFX104 significantly reduced gray mold incidence and lesion severity relative to nontreated infected plants (53–64%, detached leaves; 12–13%, whole-plant assays), achieving disease control levels similar to those obtained with the commercial biofungicide Serenade ASO^®^. Whole-genome sequencing allowed the taxonomic assignment of the NFX strains and revealed a rich repertoire of biosynthetic gene clusters and antifungal determinants. The NFX21 genome contained genes associated with N-acyl-homoserine lactone-mediated quorum-sensing and production of lipopeptides, siderophores, and extracellular lytic enzymes. The NFX104 genome harbored clusters involved in the biosynthesis of multiple siderophores, 2,4-diacetylphloroglucinol and hydrogen cyanide. Moreover, both the NFX21 and NFX104 genomes contained additional low-homology clusters that potentially encode for novel unexplored metabolites. Collectively, these results support the translational potential of NFX21 and NFX104 as biocontrol candidates for sustainable, integrated management of gray mold caused by *B. cinerea*.

## 1. Introduction

Gray mold disease caused by the necrotrophic fungus *Botrytis cinerea* poses a significant threat to global agriculture, causing pre- and post-harvest losses. *B. cinerea* infects over 1400 plant species, including high-value crops such as grapevines, strawberries, tomatoes, and ornamental plants [1,2], and is notoriously difficult to manage due to its complex and adaptive life cycle. Consequently, effective management often requires the integration of multiple strategies [3,4]. Current *B. cinerea* management strategies rely heavily on chemical fungicides, accounting for €540 million annually, representing 10% of the global fungicide market [5]. Nonetheless, overuse of chemical fungicides has led to environmental degradation, human health risks, and the emergence of resistant fungal strains [6]. This is particularly concerning for *B. cinerea*, as its short life cycle, high reproductive rate, and remarkable genetic variability facilitate the rapid development of fungicide resistance [6]. These economic and environmental challenges underscore the urgent need for sustainable alternatives.

Biological control agents (BCAs), particularly bacteria with antifungal properties, have emerged as promising and sustainable solutions to mitigate the impact of *B. cinerea* [3]. The mode of action of bacterial BCAs against fungal pathogens is diverse, involving the production of antifungal metabolites (i.e., antibiosis), competition for nutrients and space, and induction of systemic resistance in host plants [7]. Because BCAs can act through diversified and complementary mechanisms, their efficacy may be more robust across variable conditions, and their durability may be improved by reducing reliance on any single antagonistic mode of action [8]. Thus, multi-mechanistic BCAs are increasingly considered promising components of sustainable resistance management [9]. In this context, genomic mining has become a powerful tool for identifying strains with multi-layered antifungal potential. Whole-genome analyses enable the detection of biosynthetic gene clusters, lytic enzyme repertoires, secondary metabolite pathways, siderophore systems, and ISR-eliciting determinants within a single microbial candidate [10]. This systems-level insight facilitates the rational selection of BCAs with complementary and synergistic mechanisms, accelerating the development of robust biopesticides [11].

While several bacterial-based biosolutions are available for the control of gray mold, the majority rely on a limited number of genera, with *Bacillus* spp. being the most widely used [3,12]. Products such as Serenade^®^ (*Bacillus amyloliquefaciens* QST 713), Double Nickel^TM^ (*Bacillus amyloliquefaciens* D747), and Kodiak^®^ HB (*Bacillus subtilis* GB03 and *Bacillus amyloliquefaciens* QST 713) have demonstrated efficacy in reducing gray mold incidence [3,5,13]. Nonetheless, diversification of BCAs, both taxonomically and mechanistically, is essential to reduce dependency on single active strains/ingredients, strengthen integrated disease management programs, and enhance long-term durability against rapidly adapting pathogens such as *B. cinerea*.

In this study, the biological control activities against *B. cinerea* and the genomic properties of two bacterial strains, *Serratia quinivorans* NFX21 and *Pseudomonas thivervalensis* NFX104, previously isolated from the rhizosphere of plants in Portugal, are described and analyzed in detail. By integrating phenotypic assays with genome mining, we characterize their antifungal properties and ecological traits and evaluate their potential as biocontrol agents to improve the sustainability and robustness of gray mold management.

## 2. Results

### 2.1. Strains NFX21 and NFX104 Significantly Inhibited Mycelial Growth and Conidia Germination of Botrytis cinerea

To evaluate the antifungal activity of strains NFX21 and NFX104, two complementary assays were carried out: a dual (fungus–bacteria) plate assay on rich solid medium to examine the overall capacity of each bacterial strain to reduce *B. cinerea* mycelial growth and a more detailed conidia germination assay in liquid medium to quantify inhibition of spore germination and early hyphal development.

**Figure 1:**
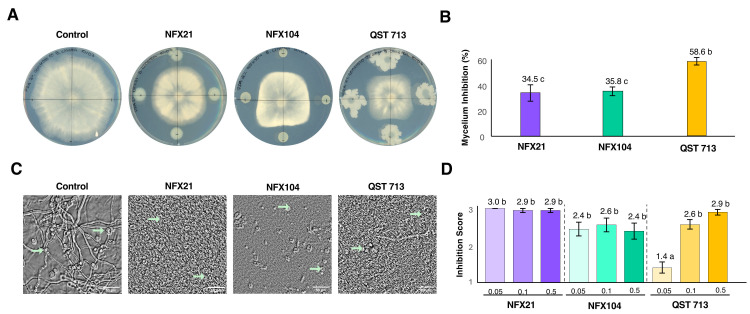
Antagonistic activities of strains NFX21, NFX104, and QST 713 against *Botrytis cinerea*. (**A**) Representative images of the *in vitro* dual-plate assay 6 days post-inoculation. (**B**) Average inhibition (%) ± SE of *B. cinerea* radial growth. Different letters indicate statistically significant differences at *p* < 0.001 according to the Kruskal–Wallis rank sum test, n = 9/treatment. (**C**) Representative images showing the inhibition of *B. cinerea* conidial germination at 48 h post-inoculation (hpi) following the different treatments. Green arrows indicate fungal spores; scale bar = 50 µm. (**D**) Mean inhibition scores ± SEs of conidial germination at 48 hpi under 0.05, 0.1, and 0.5 bacterial OD_600nm_ treatments. Score 1 = 0–30% inhibition, score 2 = 30–70% inhibition, and score 3 > 70% inhibition. Different letters indicate statistically significant differences at *p* < 0.001 according to the Kruskal–Wallis and Dunn’s *post hoc* tests, n = 18/treatment.

In the dual-plate assay, both NFX21 and NFX104 exhibited clear fungal antagonistic activity, as evidenced by the inability of *B. cinerea* to overgrow bacterial colonies and the presence of clear fungal inhibition (Figure 1A). Strains NFX21 and NFX104 significantly inhibited mycelial growth, reducing *B. cinerea* growth by 34.5% and 35.8%, respectively, relative to the nontreated fungal control (Figure 1B). The reference strain, *Bacillus amyloliquefaciens* QST 713 (the active strain in the Serenade ASO^®^ product), also showed a strong inhibitory effect against *B. cinerea*, with a 58.6% reduction in *B. cinerea* mycelial growth (Figure 1B). Although strains NFX21 and NFX104 presented lower inhibition percentages than those observed for strain QST 713, the consistent suppression across replicates suggests stable antagonistic activity.

The conidia germination assay revealed that in the nontreated control (Figure 1C), fungal conidia readily germinated and formed hyphal networks by 48 h post-inoculation (hpi). In contrast, the presence of NFX21 and NFX104 markedly inhibited germination and early development (Figure 1C). Both bacterial strains significantly suppressed conidia germination at all tested concentrations (optical density at 600 nm, OD_600nm_ = 0.05, 0.1 and 0.5), resulting in a strong overall reduction in germination relative to the control (Figure 1D). In contrast, *B. amyloliquefaciens* QST 713 showed a marked decrease in efficacy at the lowest density (OD_600nm_ = 0.05). Nonetheless, when applied at higher concentrations (OD_600nm_ = 0.1 and 0.5), the strain matched the performance of the NFX strains (Figure 1D).

### 2.2. Strains NFX21 and NFX104 Significantly Reduced Gray Mold Incidence in Tomato Detached-Leaf and Whole-Plant Pot Assays

To validate the antagonistic activities of strains NFX21 and NFX104 against *B. cinerea* infection *in planta*, we performed a tomato detached-leaf assay and a pot assay using whole tomato plants. In the detached-leaf assay, the *Botrytis*-induced lesion development was assessed 6 days post-fungal inoculation, whereas in the pot assay, gray mold symptom development was analyzed 28 days post-*B. cinerea* inoculation. In both assays, the efficacy of NFX21 and NFX104 was benchmarked against the commercial biofungicide Serenade ASO^®^.

**Figure 2:**
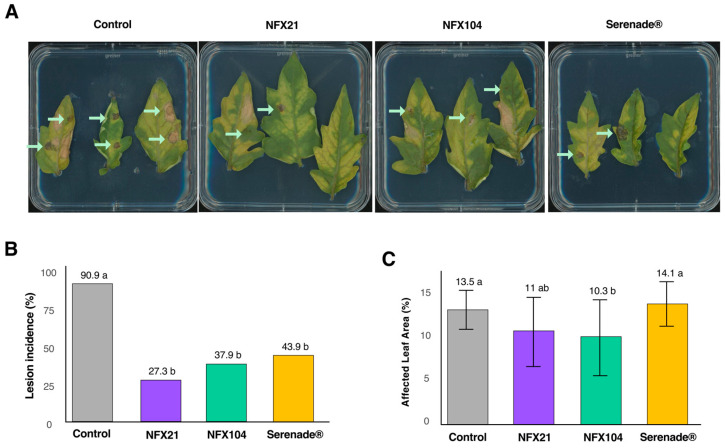
Antagonistic activity of strains NFX21, NFX104, and Serenade ASO^®^ against *Botrytis cinerea* infection in detached tomato leaves. (**A**) Representative images from the detached-leaf assay, showing the effects of the treatments 6 days post-infection. (**B**) Gray mold lesion incidence (%) per treatment. Different letters indicate statistically significant differences at *p* < 0.001, based on the Fisher exact test for count data, n = 66/treatment. (**C**) Mean of affected leaf area (%) ± SE per treatment. Different letters indicate statistically significant differences at *p* < 0.05 according to the Kruskal–Wallis rank sum test, n = 11–32/treatment. Green arrows represent *Botrytis*-induced lesions.

In the detached-leaf assay, all bacterial treatments (NFX21, NFX104, and Serenade ASO^®^) significantly reduced gray mold incidence relative to the nontreated infected control (Figure 2A). In the nontreated infected control, disease incidence reached approximately 91%, indicating widespread *B. cinerea* infection. In contrast, leaves treated with NFX21 and NFX104 showed markedly lower incidence values of about 27% and 38%, respectively, while Serenade ASO^®^ reduced disease incidence to ~44%, indicating comparable overall protection across the bacterial treatments (Figure 2B). When disease severity was estimated as the percentage of affected leaf area (%), only the application of NFX104 resulted in a significant reduction (~3%) relative to the nontreated infected control (Figure 2C). The application of NFX21 and Serenade ASO^®^ did not differ significantly from the nontreated infected control (Figure 2C).

**Figure 3:**
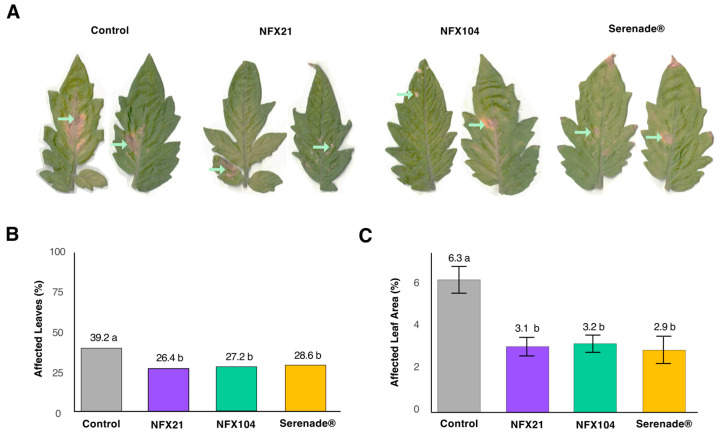
Antagonistic activity of strains NFX21, NFX104, and Serenade ASO^®^ against *Botrytis cinerea* infection in whole tomato plants under growth chamber conditions. (**A**) Representative images of gray mold-infected leaves showing treatment effects 28 days post-infection. (**B**) Gray mold lesion incidence (%) per treatment. Different letters indicate statistically significant differences at *p* < 0.001, based on the Fisher exact test for count data, n = 10/treatment. (**C**) Mean affected leaf area (%) ± SE per treatment. Different letters indicate statistically significant differences at *p* < 0.001, according to the Kruskal–Wallis rank sum test, n = 10/treatment. Green arrows represent *Botrytis*-induced lesions.

In the pot assay, the application of bacterial strains and Serenade ASO^®^ significantly decreased both disease incidence, measured as the proportion of infected leaves, and lesion severity compared with the nontreated infected control (Figure 3A–C). Disease incidence in the nontreated infected control was 39% and was significantly reduced (by 10–13%) in plants treated with strain NFX21 (26% disease incidence), NFX104 (27% disease incidence), and Serenade ASO^®^ (29% disease incidence) (Figure 3B). A similar pattern was observed for disease severity, expressed as affected leaf area (%), which decreased from 6.3% in the control to 3.2%, 2.9%, and 3.1% in plants treated with NFX21, NFX104, and Serenade ASO^®^, respectively (Figure 3C).

### 2.3. Genomic Characterization of Strains NFX21 and NFX104

**Figure 4:**
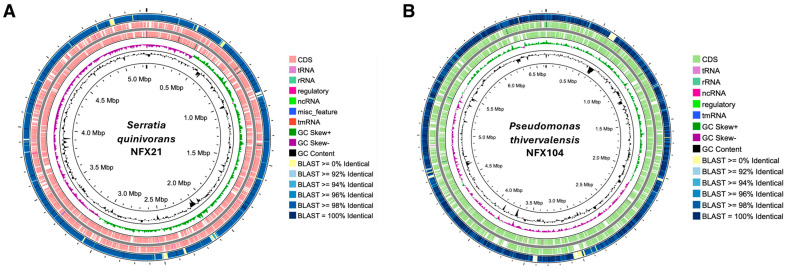
Circular view of the genomes of strains NFX21 and NFX104. (**A**) *Serratia quinivorans* NFX21 chromosome; the outer ring represents the BLASTn analysis results of the comparison between strain NFX21 and *Serratia quinivorans* NCTC11544^T^. (**B**) *Pseudomonas thivervalensis* NFX104 chromosome; the outer ring represents the BLASTn analysis results of the comparison between strain NFX104 and *P. thivervalensis* DSM 13194^T^.

To identify strains NFX21 and NFX104 and to uncover genetic features underlying their antifungal activities, their complete genomes were sequenced and analyzed in detail (Table 1, Figure 4A,B). The NFX21 genome was composed of a single circular chromosome of 5,243,286 bp, with an average GC content of 55.2% (Figure 4A). The NFX21 genome contained 4897 genes, of which 4746 were annotated as protein-coding sequences (CDSs) and 117 as RNA-related genes. Functional annotation using GHOSTKOALA assigned 3200 CDSs (67.9%) to different KEGG categories (Table 1). Phylogenomic analysis revealed that strain NFX21 genome presented high identity to the genome of *Serratia quinivorans* NCTC11544^T^ (90.3% digital DNA-DNA Hybridization (dDDH); 98.65% Average Nucleotide Identity (ANI)), supporting the classification of strain NFX21 as a member of the *S. quinivorans* species. Consistent with this, BLASTn analysis confirmed the high similarity between the NFX21 and NCTC11544^T^ genomes (Figure 4A). The genome sequence of strain NFX104 was also composed of a single circular chromosome of 6,633,357 bp with an average GC content of 61.2% (Figure 4B). A total of 5879 genes were identified in the chromosome, of which 5714 were annotated as CDSs (with protein) and 85 were RNA-related genes. GHOSTKOALA analysis resulted in the functional annotation of 3166 CDSs (54.8%) (Table 1). Phylogenomic placement revealed high identity to the type strain *Pseudomonas thivervalensis* DSM 13194^T^ (89.7% dDDH; 98.95% ANI), thus confirming strain NFX104 as a member of the *Pseudomonas thivervalensis* species.

Functional analysis indicated that both strains possessed broad metabolic potential, with the highest numbers of KEGG assignments in environmental information processing, signaling/cellular processes, genetic information processing, and central metabolism (carbohydrate and amino acid metabolism) (Table 1), consistent with metabolically versatile plant-associated bacteria. *Serratia quinivorans* NFX21 was relatively enriched in carbohydrate metabolism, nucleotide metabolism, and signaling/cellular process categories, whereas *P. thivervalensis* NFX104 showed higher representation in environmental information processing, amino acid metabolism, energy metabolism, cellular processes, cofactor/vitamin metabolism, and xenobiotics biodegradation, suggesting stronger capacities for environmental sensing/adaptation and metabolic flexibility.

Genomic functional analysis focusing on antibiotic resistance, toxin biosynthesis and secretion system genes also revealed the different genetic determinants of the NFX strains. The genome of strain NFX21 harboured four main antibiotic resistance genes: *strB* (streptomycin 6-kinase), *catB* (chloramphenicol O-acetyltransferase type B), *qnr* (fluoroquinolone resistance protein) and *vanX* (zinc D-Ala-D-Ala dipeptidase). In addition, several multidrug transporters/efflux systems (*mdfA*, *smvA*, *mexAB*, *oprM*, *cusS*, and *cusR*) were also identified. The strain NFX21 chromosome also contained homologous genes related to the type II membrane-damaging toxins, including *shlAB* genes (hemolysin and hemolysin activation/secretion protein), *hlyIII* (hemolysin III) and *plc* (phospholipase C). Moreover, multiple secretion and surface-structure systems, including *sec*, *tat*, type I, II, and VI secretion-related components; type IV pilus/competence-associated proteins; and chaperone-usher fimbrial assembly genes, were also detected in the chromosome of strain NFX21. The genome of *P. thivervalensis* NFX104 contained *mprF*, encoding phosphatidylglycerol lysyltransferase associated with reduced susceptibility to cationic antimicrobial peptides, and mostly contained multidrug transporters/efflux systems (*mdfA*, *mexAB*, *mexLJK*, *oprM*, *oprN*, *cusS*, and *cusR*). The genome also harboured an *hlyIII* (hemolysin III) homolog and four genes encoding insecticidal toxin complex protein TccC. The *sec*, *tat*, type I, II, III, and VI secretion-related components were detected in the strain NFX104 chromosome.

### 2.4. Genomic Insights into the Antifungal Features of Serratia Quinivorans NFX21

The antagonistic features of *S. quinivorans* NFX21 and *P. thivervalensis* NFX104 against *B. cinerea* suggest that both strains may produce antifungal metabolites and/or deploy lytic enzymes that impair fungal development. Based on this, we performed genome mining focusing on secondary metabolite biosynthetic gene clusters (BGCs) and other candidate antifungal determinants. AntiSMASH analysis of strain NFX21 predicted multiple BGCs, including a homoserine lactone biosynthesis gene, two Non-Ribosomal Peptide Synthetase (NRPS) clusters, two NRPS-like clusters, two NRP-metallophore clusters and an NRPS-independent (NI)-siderophore cluster (Table 2). Despite their successful identification, most NRP clusters presented low similarity to characterized BGCs in the MIBIG database (Table 2), suggesting that NFX21 may harbor species-specific or previously uncharacterized biosynthetic potential.

Nonetheless, comparative analysis revealed that the NFX21 homoserine lactone biosynthesis gene (ACNT1W_02350) presented high identity (70–85% identity at the protein level) to those functionally described in *S. plymuthica* G3 (*spsI*) and *S. liquefaciens* (*swrI*), being involved in the biosynthesis of N-butyrylhomoserine lactone (C4-HSL), N-pentanoylhomoserine lactone (C5-HSL), and N-hexanoylhomoserine lactone (C6-HSL). Moreover, the NI-siderophore cluster presented homology to the aerobactin BGC from *Xenorhabdus szentirmaii* DSM 16338 [14]. The NFX21 NRP gene ACNT1W_24085 encoded a protein highly similar (77.4% identity, 99.62% query coverage) to *S. marcescens* SwrW (BAD60917.1), which is involved in the production of the lipopeptide biosurfactant serrawetin. In addition to the identified BGCs, the NFX21 genome also harbored several genes encoding enzymes and secreted factors with potential antifungal relevance. Four chitinase-encoding genes were identified, including two predicted extracellular chitinases (ACNT1W_02855 and ACNT1W_15895), together with several genes encoding serralysins (ACNT1W_03245, ACNT1W_12740, and ACNT1W_14175) and additional extracellular proteases (ACNT1W_01090, ACNT1W_1095, and ACNT1W_20420).

### 2.5. Genomic Insights into the Antifungal Features of Pseudomonas Thivervalensis NFX104

For *P. thivervalensis* NFX104, antiSMASH analysis predicted nine BGCs, including three NRP-metallophore clusters, one NRPS cluster, one NRPS-like cluster, one hydrogen cyanide biosynthesis cluster, one type III polyketide synthase (T3PKS) cluster, one phosphonate biosynthesis cluster and one Lanthipeptide-class-II cluster (Table 3). The identified NRP-metallophore and NRPS clusters showed similarity to characterized siderophore systems, including pyoverdine biosynthesis clusters [15,16], the coelibactin siderophore biosynthesis cluster, and the *P. thivervalensis* histicorrugatin biosynthesis cluster [17] (Table 3). Importantly, the T3PKS gene identified in the *P. thivervalensis* NFX104 genome encoded a protein that showed high identity to the PhlD protein from *P. ogarae* F113, a key enzyme in 2,4-diacetylphloroglucinol (DAPG) biosynthesis (Table 3). Consistently, detailed analysis confirmed that the DAPG biosynthesis cluster (composed of the *phlACBDE* and *phlHGF* genes) in NFX104 was highly similar to the functional DAPG biosynthesis gene clusters of *P. thivervalensis* PITR2 (~99% identity) and *P. ogarae* F113 (~93% identity), both presenting antifungal activities [18,19]. Moreover, the hydrogen cyanide biosynthesis cluster from NFX104, composed of *hcnABC* genes, also presented high similarity to the functional *hcnABC* genes of *P. thivervalensis* PITR2 (~99% identity) [20]. Despite the identification of an NRPS-like gene, a phosphonate biosynthesis cluster and one lanthipeptide-class-II cluster in the genome of strain NFX104, their homology to described functional clusters was low (Table 3), suggesting that NFX104 may also encode pathways for novel bioactive compounds.

## 3. Discussion

The growing demand for sustainable agriculture has accelerated the search for innovative biological control agents to manage pathogens such as *B. cinerea*. Given its wide host range, high genetic plasticity, and increasing risk of fungicide resistance [21], identifying novel antagonistic agents with diversified modes of action is increasingly important. In this work, two plant-associated bacterial strains, *S. quinivorans* NFX21 and *P. thivervalensis* NFX104, were evaluated as novel promising candidates for biological control of *B. cinerea*, with functional activity validated through complementary *in vitro* and *in planta* assays. Both NFX strains significantly inhibited mycelial growth of *B. cinerea in vitro* (~35%) and strongly suppressed fungal conidia germination. *In planta* assays showed that the NFX strains significantly reduced gray mold incidence in detached-leaf and pot experiments, achieving 53% to 64% and 12% to 13% reductions, respectively. These effects were comparable to those obtained with the commercial product Serenade ASO^®^, which reduced incidence by 47% in detached leaves and 10% in the pot assay. Collectively, these outcomes align with previous reports describing the antagonistic activity of *Serratia* strains, including *S. quinivorans* [22,23,24], and *P. thivervalensis* against *B. cinerea* and other fungal pathogens [25,26,27], further supporting their biocontrol potential in agricultural applications. The markedly lower disease reduction induced by the tested bacteria in whole-plant pot assays compared with detached-leaf assays likely reflects the greater biological and environmental complexity of the pot system. In this sense, host physiology and tissue-level differences in intact plants may influence both pathogen infection dynamics and bacterial colonization.

In-depth genomic analysis of both NFX strains confirmed their taxonomic placement and highlighted candidate mechanisms involved in antifungal activity. For *S. quinivorans* NFX21, the predicted homoserine lactone biosynthesis locus suggests a prominent role for quorum-sensing mechanisms, potentially involving the production of multiple N-acyl-homoserine lactones (C4, C5, and C6-HSL), as reported for other *Serratia* strains [23,28,29]. In *Serratia*, N-acyl-homoserine lactones are important regulators of gene expression and control the production of metabolites and extracellular enzymes with direct antagonistic relevance, including compounds such as HCN and lytic enzymes such as chitinases [30,31,32]. In *S. plymuthica* HRO-C48, disruption of HSL signaling abolishes both root colonization capacity and the induction of systemic resistance against gray mold in bean and tomato, indicating that HSL-mediated regulation can also contribute to plant immunity [28]. Consistent with these observations, the NFX21 genome contained several genes encoding lytic enzymes (e.g., chitinase and serralysins) previously linked to fungal antagonism in *Serratia* [24,33,34]. In addition, NFX21 harbored several BGCs involved in lipopeptide production, including a gene with high similarity to *Serratia swrW* that is involved in serrawetin biosynthesis. Serrawetin contributes to *Serratia* spp. surface colonization and may also support antimicrobial activities via membrane-disrupting effects [35,36]. Together, these data suggest that NFX21 activity is likely shaped by combined contributions of quorum-sensing-regulated traits, secreted lytic enzymes, and bioactive metabolites impacting fungal growth and development.

For *P. thivervalensis* NFX104, genome mining highlighted key BGCs associated with well-characterized antifungal determinants, including DAPG and HCN. In fluorescent *Pseudomonas*, DAPG is a major contributor to disease-suppressive soils and root defense, with *phlACBD* mutants or naturally non-producing genotypes exhibiting markedly reduced biocontrol activity [37,38,39,40]. For example, *P. fluorescens* NBC275 transposon mutants in *phlD* or *phlC* lost the ability to produce DAPG and displayed drastically reduced antifungal activity against *B. cinerea* both *in vitro* and *in planta* [40]. The production of HCN by plant-associated *Pseudomonas* spp. has also been widely implicated in biocontrol, as this volatile secondary metabolite can inhibit the respiration and growth of a broad range of phytopathogenic fungi and oomycetes [41,42,43]. In several fluorescent *Pseudomonas* strains, *hcnABC* mutants or naturally non-cyanogenic isolates display reduced antagonism toward target pathogens and diminished disease suppression in host plants, supporting an additive or synergistic contribution of HCN alongside other metabolites, such as DAPG, phenazines, and cyclic lipopeptides, to overall biocontrol performance [20,44]. Interestingly, our analysis also revealed the presence of additional BGCs in the genomes of both NFX21 and NFX104 that, although detected, showed lower sequence identity to characterized functional BGCs in the MIBIG database. Several of these clusters seem to be involved in the biosynthesis of antibiotics/lipopeptides/phosphonates which may also contribute to the antifungal activities of the NFX strains. New studies are necessary to ascertain the functional contribution of these candidate clusters in shaping the antifungal activities of *S. quinivorans* and *P. thivervalensis*, including expression profiling under confrontation conditions, mutagenesis of key biosynthetic genes, and chemical characterization of secreted metabolites.

Additionally, the NFX strain genomes not only encoded the potential to produce secondary metabolites but also other traits commonly associated with plant-associated biocontrol bacteria, combining strong colonization potential (type IV pili/competence and other surface-structure genes) with multiple secretion pathways (sec/tat and type I/II/VI, in addition to type III in NFX104) that can support extracellular enzyme delivery, antimicrobial factor export, and interbacterial competition in the rhizosphere [45]. In particular, the presence of a type VI secretion system in both strains suggests the capacity to outcompete other microbes, including phytopathogens [46], while NFX104 also carries insecticidal toxin complex genes (TccCs), indicating possible dual activity against microbial pathogens and insect pests [47]. Nevertheless, some bacterial genetic features such as the prevalence of genes encoding hemolysin/phospholipase-like toxins and multiple antibiotic resistance/efflux determinants (especially in NFX21) also highlight the need for careful safety and risk assessment (non-target effects and mobility of resistance loci) before considering agricultural deployment as seed/soil inoculants or components of microbial consortia.

By integrating functional validation and genomic analysis, this study identified *Serratia quinivorans* NFX21 and *Pseudomonas thivervalensis* NFX104 as promising biocontrol agents against *B. cinerea*. Both strains demonstrated strong antifungal activity, particularly by inhibiting conidial germination and early hyphal development, and significantly reduced gray mold disease symptoms *in planta* in detached-leaf and whole-plant assays. Genome mining further indicated that the NFX strains’ antifungal activities are likely mediated by multifaceted antagonistic potential, combining biosynthetic capacity for antifungal metabolites with the production of secreted lytic enzymes and other extracellular factors.

Importantly, the level of gray mold disease suppression achieved by the NFX strains was comparable to that of an established commercial biopesticide, supporting their translational potential for integrated pest management. Future work should prioritize mechanistic validation of the predicted pathways, including targeted genetic and chemical characterization of key metabolites and enzymes, as well as performance testing across environmental conditions and production systems. Evaluating NFX21 and NFX104 both individually and in combination under greenhouse and field settings will be essential to assess their robustness, consistency, and application potential as effective gray mold biocontrol agents.

## 4. Materials and Methods

### 4.1. Bacterial Strains and Genomic Characterization

Strains NFX21 and NFX104 are part of the NFX personal collection, which contains plant- and rhizosphere-associated bacterial isolates. Strain NFX21 was isolated from the rhizosphere of *Trifolium* spp. in Besteiras, Portugal (Nascimento, unpublished results), and strain NFX104 (formerly identified as strain PLM1) was isolated from agricultural soil in Palmela, Portugal [48]. The strains were routinely cultivated in tryptic soy broth (TSB) media and maintained in glycerol stocks at −80 °C.

Genome sequencing of the NFX strains was performed by Microbes NG (https://microbesng.com/) (UK). Sequencing libraries were generated using SQK-RBK114.96. Sequencing was performed on a GridION (Oxford Nanopore Technologies, Oxford, UK) using an R10.4.1 flowcell, with basecalling performed using the r1041_e82_400bps_hac_v4.2.0 model. Reads were randomly subsampled to 50X coverage using Rasusa (V 0.7.1) [49] and assembled using Flye (V2.9.2-b1786) [50]. Assemblies were polished using Medaka (V 1.8.0) and the relevant model (r1041_e82_400bps_hac_v4.2.0). The final genome sequences were submitted to the National Center for Biotechnology Information (NCBI) database (https://www.ncbi.nlm.nih.gov/) under the accessions PRJNA1229278 (*Serratia quinivorans* NFX21) and PRJNA1154279 (*Pseudomonas thivervalensis* NFX104), and they were annotated using the NCBI prokaryotic annotation pipeline [51]. Genome circular views, BLASTn (standard parameters) and FastANI analysis against selected type strains were performed in Proksee (https://proksee.ca/) [52]. The genomic sequences of *Serratia quinivorans* NCTC11544^T^ and *Pseudomonas thivervalensis* DSM 13194^T^ were obtained from the NCBI database (accessions: GCA_900457075.1 and GCA_001269655.1, respectively). The digital DNA-DNA Hybridization (dDDH) analysis was conducted in the TYGS server (https://tygs.dsmz.de/) [53], and dDDH was calculated using the d4 formula. Functional genome annotations were performed using GHOSTKOALA [54]. Secondary metabolite clusters were identified using antiSMASH v.8.1 [55] with standard parameters (relaxed mode) and compared to the data found in the MIBiG database [56].

### 4.2. Preparation of Botrytis cinerea Spore Suspension

*Botrytis cinerea* CECT 20973 was obtained from the Spanish Type Culture Collection (CECT). The fungus was cultured for 9 days at 26 °C on potato dextrose agar (PDA). Sporulation was then induced by light shock, and the plates were covered with sterile distilled water (dH_2_O) containing 0.01% (*v*/*v*) Tween-20 using a Pasteur pipette. Colonies were gently probed with a sterile loop to release the spores, and the resulting suspension was collected with a Pasteur pipette. The suspension was filtered through a 40 µm cell strainer to separate spores from mycelial fragments. Spore concentration was determined with a Neubauer chamber and adjusted to 1 × 10^6^ spores/mL for experimental use. Unless otherwise indicated, this concentration was used throughout the experiments.

### 4.3. Dual-Plate In Vitro Assay

Strains NFX21, NFX104 and QST 713 (*Bacillus amyloliquefaciens* isolated from Serenade ASO^®^ was used as a positive control in this study) were grown overnight at 23 °C in TSB media. Bacterial suspensions were standardized to an OD_600nm_ of 0.5 in 0.03M MgSO_4_. PDA plates (2% agar) were divided into four quadrants, and a 5 µL drop of the standardized bacterial suspension was placed at 1 cm from the periphery of each quadrant, while 10 µL of the standardized *B. cinerea* spore suspension was placed at the center of each plate. Plates were incubated at 26 °C for 5 days, until the mycelium of *B. cinerea* in the nontreated controls covered the area where the bacterial spots were placed in the treated samples. Plates were then scanned for analysis, and fungal growth areas were quantified using ImageJ software v.1.54g. Fungal growth inhibition (%) was calculated by comparing the fungal growth areas under the different treatments with those of the nontreated controls. Each treatment consisted of three replicate plates with four bacterial spots per plate. Three independent assays were performed.

### 4.4. Bacterial Effects on B. cinerea Conidia Germination

The effects of strains NFX21, NFX104 and QST 713 on the germination and development of *B. cinerea* conidia were assessed by co-culture in 1:10 (*v*/*v*) Potato Dextrose Broth (PDB, diluted in sterile dH_2_O) in a 96-well microplate. The bacterial cultures were prepared by growing each strain overnight in TSB at 26 °C with shaking at 180 rpm in a New Brunswick Innova^®^ 42R incubator. On the day of the experiment, the bacterial suspensions were adjusted to OD_600nm_ values of 0.05, 0.1, and 0.5 in 1:10 (*v*/*v*) PDB. For confrontation assays, 132 µL of each bacterial suspension was mixed with 66 µL of a *B. cinerea* spore suspension (1 × 10^6^ spores/mL), and the mixtures were loaded into the microplate wells. Control treatments consisted of *B. cinerea* incubated in 1:10 (*v*/*v*) PDB without bacterial suspensions. Each treatment was performed in technical duplicates, and three images per well were captured at 48 h post-inoculation (hpi) at 10× magnification using the Tecan Spark Cyto^®^ (Männedorf, Switzerland) instrument. Three independent assays were conducted. The inhibitory effect of the bacteria on *B. cinerea* conidial germination was evaluated using a categorical and ordinal scoring system: 0–30% inhibition (score = 1), 30–70% inhibition (score = 2), and >70% inhibition (score = 3). Scores were assigned to each captured image relative to the nontreated *B. cinerea* control. Representative images for each score category (1–3) are shown in Figure S1.

### 4.5. Tomato Detached-Leaf Assay

Tomato seeds (*Solanum lycopersicum* L., cultivar Moneymaker) were germinated and grown in 1 L pots filled with Siro Horta (Siro, Mira, Portugal) substrate in a greenhouse for approximately two months. For the detached-leaf assay, leaves were excised with a razor blade, retaining the petiole, and immediately transported on ice to the laboratory. Leaves were first washed under running tap water and then rinsed with sterile dH_2_O in a laminar flow chamber. After drying on sterile paper towels, three leaves per treatment were soaked for 30 min in 50 mL of bacterial solutions (NFX21 or NFX104, OD_600nm_ = 0.5 in 0.03 M MgSO_4_) or freshly prepared Serenade ASO^®^ solution (26 mL/L in sterile dH_2_O, according to the manufacturer’s instructions). Treated leaves were then plated on 0.7% (*w*/*v*) agar square plates (120 mm × 120 mm) and air-dried in the laminar flow chamber. Infection was performed by applying 20 µL drops of *B. cinerea* spore suspension onto each leaf (two drops per leaf). Non-infected controls were prepared similarly but without *B. cinerea* inoculation to assess treatment compatibility with leaf tissue. Plates were incubated in a growth chamber at 22 °C/18 °C (day/night), 60% relative humidity, with a 16 h/8 h (light/dark) photoperiod for six days. After incubation, leaves were scanned, and lesion incidence (%) was determined by counting the number of lesions per total inoculation spots, while disease severity was expressed as the percentage of affected leaf area (%) by measuring the lesion area relative to the total leaf area, both quantified using ImageJ software v.1.54g. Each treatment included three replicated plates, with three leaves per plate and two infection sites per leaf. Three independent assays were conducted.

### 4.6. Tomato Pot Assay in Growth Chamber

Tomato seeds (*Solanum lycopersicum* L., cultivar Moneymaker) were sown in 3 L pots filled with Composana substrate (COMPO SANA Plantação, COMPO Group, Munster, Germany) and maintained under semi-controlled conditions (22–24 °C, 60–70% relative humidity, 16 h light photoperiod) with watering twice per week. Bacterial isolates NFX21 and NFX104 were maintained on Tryptic Soy Agar (TSA) for 48 h at 26 °C. For inoculum preparation, single colonies were transferred to TSB and incubated overnight at 26 °C with shaking at 150 rpm. Cells were collected by centrifugation at 4193 *g* at room temperature, washed twice with half-strength Ringer solution, and resuspended to an OD_600nm_ = 0.5. *B. cinerea* spore suspension was prepared as previously described and adjusted to 1.0 × 10^5^ spores/mL.

A randomized block design was implemented with four treatments: water control, NFX21, NFX104, and Serenade ASO^®^ applications. Foliar applications were delivered at 25 mL per plant, with Serenade applied at 26 mL/L and NFX21 or NFX104 at an OD_600nm_ of 0.5. For each treatment, two cohorts were established: one inoculated with *B. cinerea* and one mock-inoculated, each comprising ten plants. Foliar bacteria treatments were first applied two days prior to pathogen inoculation and re-applied ten days later (i.e., eight days after *B. cinerea* inoculation). To promote infection, plants were enclosed in transparent plastic bags for 24 h before inoculation. On the day of inoculation, plants received a foliar application of the *B. cinerea* spore suspension, while mock-inoculated plants received sterile water containing 0.01% Tween 20. Plants remained enclosed for an additional 48 h after inoculation, after which the bags were removed.

At 28 days post-inoculation, affected leaves (%) were determined by counting the number of affected leaves relative to the total number of leaves:

Affected leaves (%) = (Number of affected leaves/Total number of leaves) × 100;

Disease severity (%) was expressed as the percentage of affected leaf area, measured as the lesion area relative to the total leaf area in 10 scanned affected leaves per treatment using ImageJ software v.1.54g:

Disease severity (%) = (Lesion area/Total leaf area) × 100.

### 4.7. Statistical Analysis

The inhibitory effect of the bacteria on *B. cinerea* mycelial growth was calculated using the following formula:

Percentage of Inhibition (%) = [Fungal area in not treated samples − Fungal area in treated samples]/[Fungal area in not treated samples] × 100(1)

Inhibition values (%) and disease severity (i.e., affected leaf area %) were tested for normality using the Shapiro–Wilk test, and non-parametric data were analyzed with the Kruskal–Wallis rank sum test via the agricolae R package. Disease incidence (%) was analyzed by Fisher’s exact test for count data. Conidial germination scores were analyzed using the Kruskal–Wallis test followed by Dunn’s *post hoc* test for comparisons between treatments. All analyses were performed in R Studio (version 2023.12.1). Results are presented as means ± standard errors (SEs), except for spore germination assays, where error bars represent the standard deviations (SDs), and different letters indicate statistically significant differences at *p* < 0.05.

## Figures and Tables

**Table 1:** General characteristics and functional annotations of the *Serratia quinivorans* NFX21 and *Pseudomonas thivervalensis* NFX104 genomes.

	*Serratia quinivorans* NFX21	*Pseudomonas thivervalensis* NFX104
**Genome size (bp)**	5,243,286	6,633,357
**GC%**	55.2	61.2
**CDSs**	4746	5714
**GHOSTKOALA**	3200 (67.9%)	3166 (54.8%)
Environmental Information Processing	389	456
Cellular Processes and Signaling	394	368
Genetic Information Processing	407	345
Carbohydrate Metabolism	308	265
Amino Acid Metabolism	189	226
Metabolism of Cofactors and Vitamins	143	156
Cellular Processes (Core)	119	139
Energy Metabolism	108	132
Nucleotide Metabolism	110	84
Glycan Biosynthesis and Metabolism	92	69
Lipid Metabolism	72	65
Metabolism of Other Amino Acids	41	39
Xenobiotics Biodegradation and Metabolism	20	27

**Table 2:** Biosynthetic gene clusters identified in the genome of *Serratia quinivorans* NFX21 by antiSMASH analysis.

BGC	Gene	Closest Homolog	MIBIG Cluster
**Homoserine lactone biosynthesis**	ACNT1W_02350	ACR22889.1 (85.8% id, 100% qc)	n.a
**NRPS-like**	ACNT1W_04490	AAO23334.1 (35% id, 100% qc)	BGC0000397.5 Nostocyclopeptide A2
**NRPS**	ACNT1W_04735	AQX14499.1 (35% id, 100% qc)	BGC0001672.3 Monobactam
**NI-siderophore**	ACNT1W_06575	CDL83405.1 (75% id, 97.4% qc)	BGC0001498.5 Aerobactin
**NRP-metallophore**	ACNT1W_06850	AAK95846.1 (51% id, 97.9% qc)	BGC0002495.2 Photobactin
**NRPS-like**	ACNT1W_10635	TXD00259.1 (51% id, 97.9% qc)	BGC0001877.4 Cyphomycin
**NRP-metallophore**	ACNT1W_19570	AAQ59905.1 (47% id, 100% qc)	BGC0002680.2 Viobactin
**NRPS**	ACNT1W_24085	AKJ29410.1 (51% id, 79.8% qc)	BGC0001608.4 Glidopeptin

id = identity, qc = query coverage

**Table 3:** Biosynthetic gene clusters identified in the genome of *P. thivervalensis* NFX104 by antiSMASH analysis.

BGC	Genes	Closest Homolog	MIBIG Cluster
**NRP-metallophore**	ACEQ4A_04970-04980	QBQ12465.1 (74% id, 100% qc) QBQ12464.1 (82% id, 100% qc) QBQ12463.1 (84% id, 100% qc)	BGC0002693 Pyoverdine SXM-1
**Hydrogen cyanide**	ACEQ4A_07055-7065	VVN46760.1 (79% id, 90.7% qc) VVN46770.1 (80% id, 100% qc) VVN46780.1 (90% id, 100% qc)	BGC0002345.2 Hydrogen cyanide
**NRP-metallophore**	ACEQ4A_10035-10040	CAC17499.1 (47% id, 100% qc) CAC17500.1 (43% id, 100% qc)	BGC0000324.5 Coelibactin
**NRP-metallophore**	ACEQ4A_11895-11925	SDF67262.1 (100% id, 100% qc) SDF67296.1 (98% id, 100% qc) SDF67324.1 (100% id, 100% qc) SDF67357.1 (98% id, 100% qc) SDF67386.1 (99% id, 100% qc) SDF67417.1 (99% id, 100% qc) SDF67478.1 (97% id, 86.9% qc)	BGC0002422.2 Histicorrugatin
**T3PKS**	ACEQ4A_12275-12300	AAM27403.1 (98% id, 100% qc)	BGC0000280.5 2,4-diacetylphloroglucinol
**NRPS**	ACEQ4A_15870	AAY93445.1 (83% id, 100% qc)	BGC0000413.5 Pf-5 pyoverdine
**Phosphonate**	ACEQ4A_17185-17190	AHL24479.1 (55% id, 93.9% qc) ABB90392.1 (60% id, 96.8% qc)	BGC0000806.5 Phosphonoglycans BGC0000904.5 FR-900098
**NRPS-like**	ACEQ4A_23570	AAO23334.1 (31% id, 83.4% qc)	BGC0000397.5 Nostocyclopeptide A2
**Lanthipeptide-class-II**	ACEQ4A_28900	BAB04174.1 (27% id, 85.6% qc)	BGC0000517.4 Haloduracin β

id = identity, qc = query coverage

## Data Availability

The NFX21 and NFX104 strains’ genome sequences were submitted to the National Center for Biotechnology Information (NCBI) database (https://www.ncbi.nlm.nih.gov/) under the accessions PRJNA1229278 (*Serratia quinivorans* NFX21) and PRJNA1154279 (*Pseudomonas thivervalensis* NFX104).

## Supplementary Materials

The following supporting information can be downloaded at https://www.mdpi.com/article/10.3390/plants15071052/s1, Figure S1: Representative images illustrating the categorical and ordinal scoring system used to evaluate the inhibitory effect of bacterial treatments on *Botrytis cinerea* conidial germination. Images are grouped by inhibition scores: score 1 (0% to 30% inhibition), score 2 (30% to 70% inhibition), and score 3 (>70% inhibition), relative to the nontreated *B. cinerea* control (left). Examples are shown for conidia incubated in 1:10 (*v*/*v*) PDB medium at 48 h post-inoculation (hpi). Scale = 50 µm.

## References

[1] Wakeham A., Langton A., Adams S., Kennedy R. (2016). Interface of the Environment and Occurrence of *Botrytis cinerea* in Pre-Symptomatic Tomato Crops. Crop Prot..

[2] Wu Z., Zhang J., Hao J., Liu P., Liu X. (2025). Understanding Efflux-Mediated Multidrug Resistance in *Botrytis cinerea* for Improved Management of Fungicide Resistance. Microb. Biotechnol..

[3] Roca-Couso R., Flores-Félix J.D., Rivas R. (2021). Mechanisms of Action of Microbial Biocontrol Agents against *Botrytis cinerea*. J. Fungi.

[4] Leroux P. (2007). Chemical Control of Botrytis and Its Resistance to Chemical Fungicides. Botrytis: Biology, Pathology and Control.

[5] Abbey J.A., Percival D., Abbey L., Asiedu S.K., Prithiviraj B., Schilder A. (2019). Biofungicides as Alternative to Synthetic Fungicide Control of Grey Mould (*Botrytis cinerea*)—Prospects and Challenges. Biocontrol Sci. Technol..

[6] Sofianos G., Samaras A., Karaoglanidis G. (2023). Multiple and Multidrug Resistance in *Botrytis cinerea*: Molecular Mechanisms of MLR/MDR Strains in Greece and Effects of Co-Existence of Different Resistance Mechanisms on Fungicide Sensitivity. Front. Plant Sci..

[7] Bonaterra A., Badosa E., Daranas N., Francés J., Roselló G., Montesinos E. (2022). Bacteria as Biological Control Agents of Plant Diseases. Microorganisms.

[8] He D.C., He M.H., Amalin D.M., Liu W., Alvindia D.G., Zhan J. (2021). Biological Control of Plant Diseases: An Evolutionary and Eco-Economic Consideration. Pathogens.

[9] Collinge D.B., Jensen D.F., Rabiey M., Sarrocco S., Shaw M.W., Shaw R.H. (2022). Biological Control of Plant Diseases—What Has Been Achieved and What Is the Direction?. Plant Pathol..

[10] Paterson J., Jahanshah G., Li Y., Wang Q., Mehnaz S., Gross H. (2017). The Contribution of Genome Mining Strategies to the Understanding of Active Principles of PGPR Strains. FEMS Microbiol. Ecol..

[11] Köhl J., Kolnaar R., Ravensberg W.J. (2019). Mode of Action of Microbial Biological Control Agents against Plant Diseases: Relevance beyond Efficacy. Front. Plant Sci..

[12] Panebianco S., Americo S., Lo Piero A.R., Cirvilleri G. (2025). Biocontrol Activity of Novel and Known Bioproducts Based on Bacillus Strains and Basic Products against *Botrytis cinerea* in Tomato Fruit. Biol. Control..

[13] Ajijah N., Fiodor A., Dziurzynski M., Stasiuk R., Pawlowska J., Dziewit L., Pranaw K. (2023). Biocontrol Potential of *Pseudomonas* Protegens ML15 against *Botrytis cinerea* Causing Gray Mold on Postharvest Tomato (*Solanum lycopersicum* Var. *Cerasiforme*). Front. Plant Sci..

[14] Hirschmann M., Grundmann F., Bode H.B. (2017). Identification and Occurrence of the Hydroxamate Siderophores Aerobactin, Putrebactin, Avaroferrin and Ochrobactin C as Virulence Factors from Entomopathogenic Bacteria. Environ. Microbiol..

[15] Guo W., Li F., Xia J., Wang W. (2021). Complete Genome Sequence of a Marine-Derived Bacterium *Pseudomonas* Sp. SXM-1 and Characterization of Its Siderophore through AntiSMASH Analysis and with Mass Spectroscopic Method. Mar. Genomics.

[16] Hartney S.L., Mazurier S., Girard M.K., Mehnaz S., Davis E.W., Gross H., Lemanceau P., Loper J.E. (2013). Ferric-Pyoverdine Recognition by Fpv Outer Membrane Proteins of *Pseudomonas* Protegens Pf-5. J. Bacteriol..

[17] Matthijs S., Brandt N., Ongena M., Achouak W., Meyer J.M., Budzikiewicz H. (2016). Pyoverdine and Histicorrugatin-Mediated Iron Acquisition in *Pseudomonas thivervalensis*. BioMetals.

[18] Keel C., Weller D.M., Natsch A., Défago G., Cook R.J., Thomashow L.S. (1996). Conservation of the 2,4-Diacetylphloroglucinol Biosynthesis Locus among *Fluorescent Pseudomonas* Strains from Diverse Geographic Locations. Appl. Environ. Microbiol..

[19] Redondo-Nieto M., Barret M., Morrissey J., Germaine K., Martínez-Granero F., Barahona E., Navazo A., Sánchez-Contreras M., Moynihan J.A., Muriel C. (2013). Genome Sequence Reveals That *Pseudomonas fluorescens* F113 Possesses a Large and Diverse Array of Systems for Rhizosphere Function and Host Interaction. BMC Genomics.

[20] Ramette A., Frapolli M., Défago G., Moënne-Loccoz Y. (2003). Phylogeny of HCN Synthase-Encoding *HcnBC* Genes in Biocontrol Fluorescent Pseudomonads and Its Relationship with Host Plant Species and HCN Synthesis Ability. Mol. Plant-Microbe Interact..

[21] Elad Y., Vivier M., Fillinger S. (2016). Botrytis, the Good, the Bad and the Ugly. Botrytis—The Fungus, the Pathogen and Its Management in Agricultural Systems.

[22] Nascimento F.X., Espada M., Barbosa P., Rossi M.J., Vicente C.S.L., Mota M. (2016). Non-specific Transient Mutualism between the Plant Parasitic Nematode, *Bursaphelenchus xylophilus*, and the Opportunistic Bacterium *Serratia quinivorans* BXF1, a Plant-growth Promoting Pine Endophyte with Antagonistic Effects. Environ. Microbiol..

[23] Kamensky M., Ovadis M., Chet I., Chernin L. (2003). Soil-Borne Strain IC14 of *Serratia plymuthica* with Multiple Mechanisms of Antifungal Activity Provides Biocontrol of *Botrytis cinerea* and *Sclerotinia sclerotiorum* Diseases. Soil Biol. Biochem..

[24] Chlebek D., Grebtsova V., Piński A., Żur-Pińska J., Hupert-Kocurek K. (2022). Genetic Determinants of Antagonistic Interactions and the Response of New Endophytic Strain *Serratia quinivorans* KP32 to Fungal Phytopathogens. Int. J. Mol. Sci..

[25] Nascimento F.X., Urón P., Glick B.R., Giachini A., Rossi M.J. (2021). Genomic Analysis of the 1-Aminocyclopropane-1-Carboxylate Deaminase-Producing *Pseudomonas thivervalensis* SC5 Reveals Its Multifaceted Roles in Soil and in Beneficial Interactions with Plants. Front. Microbiol..

[26] Meng Y., Li J., Yuan W., Liu R., Xu L., Huang L. (2024). *Pseudomonas thivervalensis* K321, a Promising and Effective Biocontrol Agent for Managing Apple Valsa Canker Triggered by Valsa Mali. Pestic. Biochem. Physiol..

[27] Liu T., Zhang J., Wang T., Li Z., Liang H., Jiang C., Tang H., Gao J., Jiang Y., Chen C. (2024). The Novel *Pseudomonas thivervalensis* Strain JI6 Promotes Growth and Controls Rusty Root Rot Disease in *Panax ginseng*. Biol. Control..

[28] Pang Y., Liu X., Ma Y., Chernin L., Berg G., Gao K. (2008). Induction of Systemic Resistance, Root Colonisation and Biocontrol Activities of the Rhizospheric Strain of *Serratia plymuthica* Are Dependent on N-Acyl Homoserine Lactones. Eur. J. Plant Pathol..

[29] Eberl L., Winson M.K., Sternberg C., Stewart G.S.A.B., Christiansen G., Chhabra S.R., Bycroft B., Williams P., Molin S., Givskov M. (1996). Involvement of *N*-acyl-L-homoserine Lactone Autoinducers in Controlling the Multicellular Behaviour of *Serratia liquefaciens*. Mol. Microbiol..

[30] Liu X., Bimerew M., Ma Y., Müller H., Ovadis M., Eberl L., Berg G., Chernin L. (2007). Quorum-Sensing Signaling Is Required for Production of the Antibiotic Pyrrolnitrin in a Rhizospheric Biocontrol Strain of *Serratia plymuthica*. FEMS Microbiol. Lett..

[31] Liu X., Jia J., Popat R., Ortori C.A., Li J., Diggle S.P., Gao K., Cámara M. (2011). Characterisation of Two Quorum Sensing Systems in the Endophytic *Serratia plymuthica* Strain G3: Differential Control of Motility and Biofilm Formation According to Life-Style. BMC Microbiol..

[32] Van Houdt R., Moons P., Aertsen A., Jansen A., Vanoirbeek K., Daykin M., Williams P., Michiels C.W. (2007). Characterization of a *LuxI*/*LuxR*-Type Quorum Sensing System and *N*-Acyl-Homoserine Lactone-Dependent Regulation of Exo-Enzyme and Antibacterial Component Production in *Serratia plymuthica* RVH1. Res. Microbiol..

[33] Zarei M., Aminzadeh S., Zolgharnein H., Safahieh A., Daliri M., Noghabi K.A., Ghoroghi A., Motallebi A. (2011). Characterization of a Chitinase with Antifungal Activity from a Native *Serratia marcescens* B4A. Braz. J. Microbiol..

[34] Moon C., Seo D.J., Song Y.S., Hong S.H., Choi S.H., Jung W.J. (2017). Antifungal Activity and Patterns of *N*-Acetyl-Chitooligosaccharide Degradation via Chitinase Produced from *Serratia marcescens* PRNK-1. Microb. Pathog..

[35] Li H., Tanikawa T., Sato Y., Nakagawa Y., Matsuyama T. (2005). *Serratia marcescens* Gene Required for Surfactant Serrawettin W1 Production Encodes Putative Aminolipid Synthetase Belonging to Nonribosomal Peptide Synthetase Family. Microbiol. Immunol..

[36] Shanks R.M.Q., Stella N.A., Lahr R.M., Wang S., Veverka T.I., Kowalski R.P., Liu X. (2012). Serratamolide Is a Hemolytic Factor Produced by *Serratia marcescens*. PLoS ONE.

[37] de Souza J.T., Weller D.M., Raaijmakers J.M. (2003). Frequency, Diversity, and Activity of 2,4-Diacetylphloroglucinol-Producing Fluorescent *Pseudomonas* Spp. in Dutch Take-All Decline Soils. Phytopathology.

[38] Weller D.M., Landa B.B., Mavrodi O.V., Schroeder K.L., De La Fuente L., Blouin Bankhead S., Allende Molar R., Bonsall R.F., Mavrodi D.V., Thomashow L.S. (2007). Role of 2,4-Diacetylphloroglucinol-Producing Fluorescent *Pseudomonas* Spp. in the Defense of Plant Roots. Plant Biol..

[39] Raaijmakers J.M., Weller D.M. (1998). Natural Plant Protection by 2,4-Diacetylphloroglucinol-Producing *Pseudomonas* Spp. in Take-All Decline Soils. Mol. Plant-Microbe Interact..

[40] Dutta S., Yu S.-M., Lee Y.H. (2020). Assessment of the Contribution of Antagonistic Secondary Metabolites to the Antifungal and Biocontrol Activities of *Pseudomonas fluorescens* NBC275. Plant Pathol. J..

[41] Blumer C., Haas D. (2000). Mechanism, Regulation, and Ecological Role of Bacterial Cyanide Biosynthesis. Arch. Microbiol..

[42] Voisard C., Keel C., Haas D., Dèfago G. (1989). Cyanide Production by *Pseudomonas fluorescens* Helps Suppress Black Root Rot of Tobacco under Gnotobiotic Conditions. EMBO J..

[43] Nandi M., Selin C., Brawerman G., Fernando W.G.D., de Kievit T. (2017). Hydrogen Cyanide, Which Contributes to *Pseudomonas chlororaphis* Strain PA23 Biocontrol, Is Upregulated in the Presence of Glycine. Biological Control.

[44] Michelsen C.F., Stougaard P. (2012). Hydrogen Cyanide Synthesis and Antifungal Activity of the Biocontrol Strain *Pseudomonas fluorescens* In5 from Greenland Is Highly Dependent on Growth Medium. Can. J. Microbiol..

[45] Ramey B.E., Koutsoudis M., Bodman S.B.V., Fuqua C. (2004). Biofilm Formation in Plant–Microbe Associations. Curr. Opin. Microbiol..

[46] Bernal P., Llamas M.A., Filloux A. (2017). Type VI Secretion Systems in Plant-associated Bacteria. Environ. Microbiol..

[47] Flury P., Aellen N., Ruffner B., Péchy-Tarr M., Fataar S., Metla Z., Dominguez-Ferreras A., Bloemberg G., Frey J., Goesmann A. (2016). Insect Pathogenicity in Plant-Beneficial Pseudomonads: Phylogenetic Distribution and Comparative Genomics. ISME J..

[48] Nascimento F.X., Glick B.R., Rossi M.J. (2019). Isolation and Characterization of Novel Soil- and Plant-Associated Bacteria with Multiple Phytohormone-Degrading Activities Using a Targeted Methodology. Access Microbiol..

[49] Hall M. (2022). Rasusa: Randomly Subsample Sequencing Reads to a Specified Coverage. J. Open Source Softw..

[50] Kolmogorov M., Yuan J., Lin Y., Pevzner P.A. (2019). Assembly of Long, Error-Prone Reads Using Repeat Graphs. Nat. Biotechnol..

[51] Angiuoli S.V., Gussman A., Klimke W., Cochrane G., Field D., Garrity G.M., Kodira C.D., Kyrpides N., Madupu R., Markowitz V. (2008). Toward an Online Repository of Standard Operating Procedures (SOPs) for (Meta)Genomic Annotation. OMICS A J. Integr. Biol..

[52] Grant J.R., Enns E., Marinier E., Mandal A., Herman E.K., Chen C., Graham M., Van Domselaar G., Stothard P. (2023). Proksee: In-Depth Characterization and Visualization of Bacterial Genomes. Nucleic Acids Res..

[53] Meier-Kolthoff J.P., Carbasse J.S., Peinado-Olarte R.L., Göker M. (2021). TYGS and LPSN: A Database Tandem for Fast and Reliable Genome-Based Classification and Nomenclature of Prokaryotes. Nucleic Acids Res..

[54] Kanehisa M., Sato Y., Morishima K. (2016). BlastKOALA and GhostKOALA: KEGG Tools for Functional Characterization of Genome and Metagenome Sequences. J. Mol. Biol..

[55] Blin K., Shaw S., Augustijn H.E., Reitz Z.L., Biermann F., Alanjary M., Fetter A., Terlouw B.R., Metcalf W.W., Helfrich E.J.N. (2023). AntiSMASH 7.0: New and Improved Predictions for Detection, Regulation, Chemical Structures and Visualisation. Nucleic Acids Res..

[56] Zdouc M.M., Blin K., Louwen N.L.L., Navarro J., Loureiro C., Bader C.D., Bailey C.B., Barra L., Booth T.J., Bozhüyük K.A.J. (2025). MIBiG 4.0: Advancing Biosynthetic Gene Cluster Curation through Global Collaboration. Nucleic Acids Res..

