# Supplementary material for: Functional and Genomic Characterization of Serratia quinivorans NFX21 and Pseudomonas thivervalensis NFX104, Novel Biocontrol Agents Against Botrytis cinerea
Source: Plants (Basel). 2026 Mar 29;15(7):1052. doi: 10.3390/plants15071052 (PMC13074693; doi:10.3390/plants15071052)
Supplement: Supplementary file 1 [file plants-15-01052-s001.zip › plants-4161229-supplementary.pdf]

**Functional and Genomic Characterization of *Serratia quinivorans* NFX21 and *Pseudomonas thivervalensis* NFX104, Novel Biocontrol Agents Against *Botrytis cinerea***

# Supplementary Data

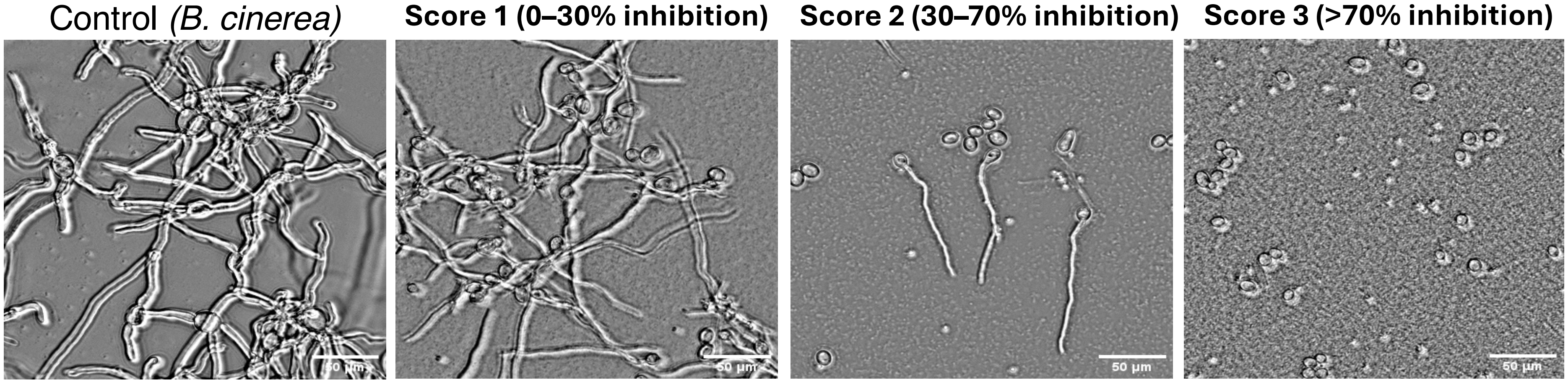


**Figure S1**- Figure S1- Representative images illustrating the categorical and ordinal scoring system used to evaluate the inhibitory effect of bacterial treatments on *Botrytis cinerea* conidial germination. Images are grouped by inhibition scores: score 1 (0% to 30% inhibition), score 2 (30% to 70% inhibition), and score 3 (>70% inhibition), relative to the nontreated B. cinerea control (left). Examples are shown for conidia incubated in 1:10 (v/v) PDB medium at 48 h post-inoculation (hpi). Scale = 50 µm.
